# Tell me how much your opponent team runs and I will tell you how much you should run: A predictive model applied to Spanish high-level football

**DOI:** 10.5114/biolsport.2024.132984

**Published:** 2023-12-19

**Authors:** Julen Castellano, Roberto López-Del Campo, Raúl Hileno

**Affiliations:** 1 GIKAFIT research Group, Department of Physical Education and Sport, University of the Basque Country, (UPV/EHU), Vitoria-Gasteiz, Spain; 2 Department of Competitions and Mediacoach, LaLiga, Madrid, Spain; 3 INEFC Lleida, University of Lleida, Lleida, Spain

**Keywords:** Match analysis, Time-motion, Team sports, Regression analysis, Situational variables

## Abstract

The aim of this study was to predict a team’s accumulated distance (TotDisTea) and accumulated distance at > 21 km/h (TotDis21Tea) in the Spanish Football First Division. 2,946 team physical performances (out of 3040 possible) during four seasons (from 2016–17 to 2019–20) were analysed. The outcome variables were the TotDisTea and TotDis21Tea when the ball was in play. Eight predictor variables were used: the distance accumulated and accumulated at > 21 km/h by the opponent (TotDisOpp and TotDis21Opp) were registered in km, the effective playing (EffPlaTim) and possession (BalPos) time were recorded in min, match location (MatLoc) had two levels (home and away), match outcome (MatOut) had three levels (lost, drawn, and won), and the teams were grouped in four levels (Champions League, Europa League, remained, and relegation) distinguishing the observed team (TeaLev) and the opponent team (OppLev) in the match. A total of 127 models were estimated from the all-possible regressions procedure for each outcome variable. The model with six predictor variables was selected as the best model to predict the TotDisTea (*R^2^_adj_* = .82). The predictor variables TotDisOpp, EffPlaTim, and BalPos had a greater contribution to the mean outcome value than the predictors OppLev, TeaLev, and MatLoc. All models estimated to predict TotDis21Tea had little predictive power (*R^2^_adj_* < .38). The findings of this study have both theoretical and practical implications for practitioners. The interaction between teams has a great effect on the conditional response. Before the match, teams could use this information to anticipate the physical demand expected in the next match, and after the match, be able to assess whether the physical response was similar to expected, and make decisions.

## INTRODUCTION

Effective playing time in football refers to the actual amount of time the ball is in play during a match, excluding stoppages for events such as fouls, injuries, substitutions, and other breaks in play. It represents the duration in which the game is actively progressing. More than a decade ago, it was proposed that effective playing time strongly conditions the physical response of professional football players in competition. Usually, the effective playing time accounts for a little over 50% of the total match time. Regarding external load, close to 70% of the running performance is accumulated in this period of play, being able to reach more than 95% when it comes to high-speed running [1]. In domestic league matches, only in effective game time can teams score or concede a goal. Above all, at this moment of the game, players are especially active. This does not prevent teams from making strategic use of breaks, trying to reduce stoppages when losing or lengthening them when the score is favourable [2].

Huge amounts of research have been carried out thanks to computerized tracking systems, applying the five properties under the concept of “the five Vs” [3]. One of these concepts is volume, that is, the magnitude of the data. Nowadays, due to the size of datasets in team sports, studies can be developed that span multiple leagues [4], categories [5, 6] or seasons [7]. The studies that gather thousands of individual and collective performances in their analyses address explanatory proposals based on the inclusion in the models of situational variables (e.g., match location, match outcome, level of the opponent, type of competition, etc.). Overall, nowadays, physical response is being better understood [8].

With respect to match location (e.g., home or away), the results are incomplete [9, 10], and probably influenced by other contextual variables (e.g., match outcome), which could explain the disparity of results [9]. Regarding match outcome, researchers reported that football players perform significantly less high intensity activity when winning than when losing or drawing [1, 10]. However, the category of the league in which the teams participate (e.g., the second division compared to the Spanish first division of soccer) could increase the connection between physical response and success in the competition at the end of the season [6]. The results of one recent study [8] that studied the differences between both phases of play (attack and defence) show that the distance covered by teams when they have the ball is shorter than when they do not (relativizing the physical response to each minute of ball possession or non-possession). Finally, the results of the academic literature emphasize the importance of taking into account the opponent’s level (e.g., high, medium, and low) during the assessment of the physical response of football performance [11, 12], briefly concluding that the higher the opponent’s level, the higher will be the physical demands required. However, it is unknown whether the physical performance of the opponent affects the physical response of the other team.

Despite the constant use of descriptive and explicative analytical techniques in match analysis, there are still few available studies that have developed predictive models of sports performance [13]. These types of studies have the common purpose to determine the most effective ways of playing and using multidimensional qualitative data instead of unidimensional frequency data. The ability to describe football match play has improved [14]. Previously, a study proposed the implementation of predictive models of the physical response of running in professional football [10]. The authors attempted to predict the distance players would cover when the match status changed or the team played home/away against strong/weak opposition. It seems that in the dynamics of a match, the team performance is a combination of knowing what to do, wanting to do it, and what the opponent allows one to do. Furthermore, all this is conditioned by the independent and interactive effects of fixed (e.g., match location, type of championship) and changing (e.g., match status, substitutions, injuries) situational variables.

The aim of this study was to predict the total distance covered by a football team and the total distance covered above 21 km/h from different situational variables of the competition. The predictive variables involved in this study were: the match location (home or away), the final match outcome (lost, drawn or won), the level of the team and opponent team, the effective playing time (in min), the possession of the ball (in min), and the distance accumulated by the rivals in both total distance and at > 21 km/h. The starting hypothesis is that, especially, the physical performance of rival teams conditions the physical performance of the team and vice versa, in addition to other contextual variables. The results of the present study may allow football practitioners to predict the physical response in competition depending on possible scenarios in order to prepare players/teams during the training process.

## MATERIALS AND METHODS

### Approach to the problem

Data collection was carried out during four seasons of the Spanish Football First Division (LaLiga), from season 2016–17 to 2019–20. The computerized multi-camera tracking system TRACAB® was used for recording teams’ physical performances. Several predictive linear regression models were proposed to estimate both the total distance covered and the distance running at more than 21 km/h in a match by teams considering predictor variables such as match location, distance covered by the opponent, quality of the team and opponent, time in possession, effective playing time, and match outcome.

### Subjects

2,946 teams’ performances (1,473 matches) were obtained from four seasons of the Spanish Football First Division (LaLiga), which authorised the use of the variables included in this study. The sample included 96% of the total possible performances (3,040 performances = 10 matches per match day × 2 performances of two teams × 38 match days × 4 seasons). Those matches where the information required was not available were excluded. In accordance with its ethical guidelines, this investigation does not include information that identifies football players. Data were treated in accordance with the Declaration of Helsinki and were approved by the Ethics Committee on Research on Human Beings (CEISH) of the university.

### Variables

A total of eight predictive variables and two outcome variables were recorded (Table 1).

**Table 1:** Properties of the study variables.

Role	Name	Value	Description
Predictor	Match location (MatLoc)	0 = Away	The analysed team played away from home
		1 = Home	The analysed team played at home
	Match outcome (MatOut)	1 = Lost	The analysed team lost the match
		2 = Drawn	The analysed team drawn the match
		3 = Won	The analysed team won the match
	Team level (TeaLev)	1 = Relegation	The analysed team finished between the 18^th^ and 20^th^ position
		2 = Remained	The analysed team finished between the 8^th^ and 17^th^ position
		3 = Europa League	The analysed team finished between the 5^th^ and 7^th^ position
		4 = Champions League	The analysed team finished between the 1^st^ to 4^th^ position
	Opponent’s level (OppLev)	1 = Relegation	The opposing team finished between 18^th^ and 20^th^ position
		2 = Remained	The opposing team finished between the 8^th^ and 17^th^ position
		3 = Europa League	The opposing team finished between the 5^th^ and 7^th^ position
		4 = Champions League	The opposing team finished between the 1^st^ and 4^th^ position
	Effective playing time (EffPlaTim)		Effective playing time in the match in minutes (min)
	Ball possession (BallPos)		Ball possession of the analysed team in minutes (min)
	Total distance covered by the opponent (TotDisOpp)		Total distance covered by the opposing team in kilometres (km)
	Total distance covered by the opponent at 21 km/h (TotDis21Opp)		Total distance covered at > 21 km/h by the opposing team in kilometres (km)
Outcome	Total distance covered by the team (TotDisTea)		Total distance covered by the analysed team in kilometres (km)
	Total distance covered by the team at 21 km/h (TotDis21Tea)		Total distance covered at > 21 km/h by the analysed team in kilometres (km)

*Note.* Within each variable, the category with the lowest numerical value (e.g., the category away in match location variable) was considered as the reference category in the multiple linear regression.

### Procedures

The computerized multi-camera tracking system TRACAB® (ChyronHego, New York, USA) was used to record time-motion data. The ball-possession duration was obtained by OPTA® Sportsdata Company (Opta Sports, London, UK). Both TRACAB and OPTA are managed by Mediacoach® software. The reliability of the OPTA system has been previously proved [15] and the reliability of the TRACAB video-tracking system has also been recently tested for physical demand [16, 17], showing good quality data. Generated reports were exported into Microsoft Office Excel (Microsoft Corporation, Washington, USA).

### Statistical analysis

Categorical variables were described by absolute and relative frequencies. Continuous variables were described by the mean (standard deviation) or median (interquartile interval) according to whether the assumption of normality was met or not. This assumption was verified using standardized normal probability plots (P-P plots) and histograms with normal-density plots. The descriptive analysis of the study variables was complemented with the estimation of the confidence intervals for a proportion (Wald method), mean (normal method), and median (exact binomial method).

The *all-possible regressions procedure* was used to select the best predictive linear regression model for both TotDisTea and TotDis21Tea. Nevertheless, no *stepwise regression procedure* was used because these automatic predictive selection methods often are problematic [18]. The regression procedure consists of constructing all possible sub-models by combining the predictor variables of the maximum model and assessing the degree of compliance in each one with the established selection criteria [19]. For the outcome variable TotDisTea, the initial maximum model included seven predictive variables (MatLoc, MatOut, TeaLev, OppLev, EffPlaTim, BalPos, and TotDisOpp); and for the outcome variable TotDis21Tea, the initial maximum model also included seven predictive variables (MatLoc, MatOut, TeaLev, OppLev, EffPlaTim, BalPos, and TotDis21Opp). The selection criteria established were as follows: (a) the principle of parsimony; (b) a small value of Mallow’s *C_p_*; and (c) a large value of the adjusted coefficient of determination (*R^2^_adj_*).

Once the best model to predict the TotDisTea and the TotDis21Tea was chosen, the reliability of its predictions was evaluated by crossvalidation. Next, it was determined whether this model met the following assumptions (the statistics and graphs used to test these assumptions are specified in parentheses): (a) absence of outliers and influential observations (internally and externally studentized residual, leverage, Cook’s distance, DFIT statistic, and covariance ratio); (b) absence of collinearity (variance inflation factor); (c) normality of the distribution of the residuals (normal P-P plot of internally studentized residuals); and (d) linearity of the relationship and homogeneity of variances of the residuals (scatter plot between the externally studentized residuals and the predicted values or the values of the predictor variables). After checking the diagnostics of the selected model, its parameters (*β_i_*) and its standardized regression coefficients (*beta*) were estimated. Finally, the model equation obtained was used to predict the outcome for certain value patterns of the predictive variables.

All statistical analyses were performed with Stata/IC version 17.0 software (StataCorp, College Station, TX, USA), considering a significance level of *p* ≤ .05.

## RESULTS

Table 2 shows the descriptive analysis (mean and standard deviation, median and interquartile interval, or absolute and relative frequencies) and inferential analysis (confidence intervals for a mean, median, or proportion) of the variables used to build the multiple linear regression model.

**Table 2:** Descriptive and inferential analysis of the study variables.

		95% CI	
Variable		*LL*	*UL*
Match location – *n* (%)			
Away	1534 (52.07)	50.27	53.87
Home	1412 (47.93)	46.13	49.73

Match outcome – *n* (%)			
Lost	1186 (40.26)	38.49	42.03
Drawn	602 (20.43)	18.98	21.89
Won	1158 (39.31)	37.54	41.07

Team/opponent’s level – *n* (%)			
Relegation	442 (15.00)	13.71	16.29
Remained	1466 (49.76)	47.96	51.57
Europa League	444 (15.07)	13.78	16.36
Champions League	594 (20.16)	18.71	21.61

Effective playing time (min) – *M* (*SD*)	52.75 (4.95)	52.57	52.92

Ball possession (min) – *Mdn* [*IQI*]	25.80 [22.10, 30.10]	25.50	26.10

Total distance covered by the team/opponent (km) – *M* (*SD*)	78.28 (6.35)	78.05	78.50

Total distance covered at > 21 km/h by the team/opponent (km) – *M* (*SD*)	5.79 (0.84)	2.53	10.63

*Note. M* = mean; *SD* = standard deviation; *Mdn* = median; *IQI* = interquartile interval; *n* = number of observations; CI = confidence interval for a proportion, mean, or median; *LL* = lower limit; *UL* = upper limit.

To predict TotDisTea or TotDis21Tea a total of 254 linear models (127 models for each outcome) were estimated from all the possible regression procedures. An attempt was also made to build a model for predicting TotDis21Tea, but it was ultimately not built because both the maximum model and the 126 derived sub-models had little predictive power (*R^2^_adj_* < .38).

Table 3 presents only 17 models (10 multiple and 7 single) of the 127 models estimated in total to predict TotDisTea, ordered from lowest to highest Mallow’s *C_p_*. Specifically, the model with the lowest *C_p_* (9.47) and highest *R^2^_adj_* (.8237) was the model containing six predictor variables out of the initial seven and excluding MatOut. The second model with the lowest *C_p_* (13.00) and highest *R^2^_adj_* (.8236) was the maximum model with seven predictor variables, which had a prediction loss of 0.01% with respect to the first model in the table. The rest of the models in the table had a prediction loss between 0.04 and 0.24% with respect to the first model. Thus, the model with all predictor variables except MatOut was selected as the best model to predict the TotDisTea for the following reasons: (a) model with two parameters less than the maximum model; (b) model with lower *C_p_* and higher *R^2^_adj_*; and (c) only model with a *C_p_* value lower than its number of parameters (*C_p_* < p+1), which indicated that the selected model had a lower error variability than the maximum model.

**Table 3:** Comparison between the 10 multiple models with the lowest Mallow’s *C_p_* and the seven simple models (outcome: TotDisTea).

Model	Predictors	*C_p_*	p+1	*R^2^_adj_*	*Shrinkage*
1	MatLoc TeaLev OppLev EffPlaTim BalPos TotDisOpp	9.47	11	.8237	base
2	MatLoc MatOut TeaLev OppLev EffPlaTim BalPos TotDisOpp	13.00	13	.8236	0.01%
3	TeaLev OppLev EffPlaTim BalPos TotDisOpp	15.46	10	.8233	0.04%
4	MatOut TeaLev OppLev EffPlaTim BalPos TotDisOpp	18.87	12	.8232	0.05%
5	MatLoc TeaLev EffPlaTim BalPos TotDisOpp	29.73	8	.8223	0.14%
6	MatLoc MatOut TeaLev EffPlaTim BalPos TotDisOpp	30.94	10	.8223	0.14%
7	TeaLev EffPlaTim BalPos TotDisOpp	37.14	7	.8218	0.19%
8	MatOut TeaLev EffPlaTim BalPos TotDisOpp	41.07	9	.8217	0.20%
9	MatLoc OppLev EffPlaTim BalPos TotDisOpp	46.45	8	.8213	0.24%
10	MatLoc MatOut OppLev EffPlaTim BalPos TotDisOpp	48.24	10	.8213	0.24%
…	…	…	…	…	…
64	TotDisOpp	1614.89	2	.7270	9.67%
95	EffPlaTim	3441.38	2	.6175	20.62%
111	OppLev	12469.64	4	.0762	74.75%
120	BalPos	13100.30	2	.0388	78.49%
123	TeaLev	13314.65	4	.0255	79.82%
125	MatOut	13701.66	2	-.0002	82.39%
127	MatLoc	13752.28	2	-.0003	82.40%

*Note. C_p_* = Mallow’s *C_p_*; p+1 = number of parameters of the model (including the constant); *R^2^_adj_*= adjusted coefficient of determination; *shrinkage* = prediction loss.

In relation to the reliability of the selected model, *R^2^_mean_* = .8229 was obtained from cross-validation. This result indicated that the true predictive capacity of the model when executed with external samples was around 82%.

Regarding the diagnostics of the selected model, 13 observations with studentized residuals > |3|, with leverage values > 2×(p+1)/n, with DFITs > |√((4×(p+1))⁄n)|, or with covariance ratios outside the interval 1±3×(p+1) were found. However, these outliers or influential observations were not removed from the sample because they were correctly recorded and because they were part of the reality of the game. The data did not present collinearity because the variance inflation factor was less than three in all the predictor variables. The normality assumption was met because all the internally studentized residuals were on the diagonal of the normal P-P plot; and the assumptions of linearity and homogeneity of variances were met because the externally studentized residuals did not present any defined pattern and were randomly distributed in the scatter plots.

The *F*-test of global significance revealed that the set of parameters of the selected model explained a significant part of the variability of the TotDisTea, *F*_(10,2935)_ = 1376.84, *p* < .001; and the *F*-tests of individual significance revealed that each predictor variable included in the model had a statistically significant contribution (*p* < .005) to the multiple linear regression equation (see Table 4). The *b* coefficients and their confidence intervals indicated that playing at home (relative to playing away), playing against a Champions League team (compared to playing against a relegation team), for each minute of increase in effective playing time, and for each kilometre of increase of the total distance covered by the opponent, the mean total distance, in kilometres, covered by the team increased significantly; in contrast, being a Champions League team (compared to being a relegation team) and for each minute of increased ball possession, the mean total distance covered by the team decreased significantly. For their part, the beta coefficients indicated that predictors TotDisOpp, EffPlaTim, and BalPos had a greater contribution to the TotDisTea outcome than the predictors OppLev, TeaLev, and MatLoc.

**Table 4:** Parameters of the selected model to predict the total distance covered by the team.

95% CI						
Predictors	*b*	*LL*	*UL*	*^p^t-test*	*^p^F-test*	*beta*
Match location					.005	
Away (0)	0 (base)					
Home (1)	0.284	0.087	0.480	.005		0.022

Team level					< .001	
Relegation (1)	0 (base)					
Remained (2)	-0.079	-0.364	0.206	.586		-0.006
Europa League (3)	0.486	0.131	0.842	.007		0.027
Champions League (4)	-0.633	-0.983	-0.284	< .001		-0.040

Opponent’s level					< .001	
Relegation (1)	0 (base)					
Remained (2)	0.153	-0.132	0.438	.291		0.012
Europa League (3)	0.004	-0.352	0.360	.983		< 0.001
Champions League (4)	0.751	0.402	1.100	< .001		0.047

Effective playing time (min)	0.424	0.391	0.457	< .001	< .001	0.331

Ball possession (min)	-0.271	-0.292	-0.251	< .001	< .001	-0.254

Total distance covered by the opponent (km)	0.712	0.687	0.738	< .001	< .001	0.712

Constant	7.077	5.770	8.384	< .001		

*Note. b* = regression coefficient; CI = confidence interval for parameter *β*; *LL* = lower limit; *UL* = upper limit; *p_t-test_* = *t*-test of significance of regression coefficient; *p_F-test_* = *F*-test of significance of predictor; *beta* = standardized regression coefficient.

From the *b* coefficients in Table 4, the following equation was defined to predict the mean total distance covered by the team in kilometres:

**Equation 1:** TotDisTea (km) = 7.077 + 0.284 × MatLoc1 + (-0.079 × TeaLea2 + 0.486 × TeaLev3 – 0.633 × TeaLev4) + (0.153 × OppLev2 + 0.004 × OppLev3 + 0.751 × OppLev4) + 0.424 × EffPlaTim – 0.271 × BalPos + 0.712 × TotDisOpp

In Table 5, the previous equation was applied and the mean TotDisTea was predicted for different values of the predictor variables selected with practical criteria. This equation can be applied to other different values as long as they belong to the range of values observed in the sample used to estimate the model (i.e., TeaLev and OppLev between relegation and Champions League, EffPlaTim between 36.8 and 68.2 min, BalPos between 12.4 and 49.4 min, and TotDisOpp between 52.5 and 98.0 km). For example, to predict the mean total distance covered by a Champions League team that possesses the ball 41 min in a match with an effective playing time of 65 min and plays away from home against a team that is staying up, that covers a total distance of 55 km, the equation is applied as follows:

**TABLE 5:** Prediction of the total distance covered by the team (km) for different values of match location, team level, opponent’s level, effective playing time (min), ball possession (min), and total distance covered by the opponent (km).

EffPlaTim and BalPos												
					40			50			60	
MatLoc	TeaLev	OppLev	TotDisOpp	15	25	35	15	25	35	15	25	35
			70	69.8	67.1	64.4	74.0	71.3	68.6	78.3	75.6	72.8
Away	Relegation	Relegation	80	76.9	74.2	71.5	81.2	78.4	75.7	85.4	82.7	80.0
			90	84.0	81.3	78.6	88.3	85.6	82.8	92.5	89.8	87.1

			70	70.6	67.8	65.1	74.8	72.1	69.4	79.0	76.3	73.6
Away	Relegation	Champions	80	77.7	75.0	72.2	81.9	79.2	76.5	86.1	83.4	80.7
			90	84.8	82.1	79.4	89.0	86.3	83.6	93.3	90.6	87.8

			70	69.2	66.5	63.7	73.4	70.7	68.0	77.6	74.9	72.2
Away	Champions	Relegation	80	76.3	73.6	70.9	80.5	77.8	75.1	84.8	82.0	79.3
			90	83.4	80.7	78.0	87.6	84.9	82.2	91.9	89.2	86.5

			70	69.9	67.2	64.5	74.2	71.4	68.7	78.4	75.7	73.0
Away	Champions	Champions	80	77.0	74.3	71.6	81.3	78.6	75.8	85.5	82.8	80.1
			90	84.2	81.4	78.7	88.4	85.7	83.0	92.6	89.9	87.2

			70	70.1	67.4	64.7	74.3	71.6	68.9	78.6	75.8	73.1
Home	Relegation	Relegation	80	77.2	74.5	71.8	81.4	78.7	76.0	85.7	83.0	80.3
			90	84.3	81.6	78.9	88.6	85.8	83.1	92.8	90.1	87.4

			70	70.8	68.1	65.4	75.1	72.4	69.6	79.3	76.6	73.9
Home	Relegation	Champions	80	78.0	75.2	72.5	82.2	79.5	76.8	86.4	83.7	81.0
			90	85.1	82.4	79.6	89.3	86.6	83.9	93.6	90.8	88.1

			70	69.4	66.7	64.0	73.7	71.0	68.3	77.9	75.2	72.5
Home	Champions	Relegation	80	76.6	73.9	71.1	80.8	78.1	75.4	85.0	82.3	79.6
			90	83.7	81.0	78.3	87.9	85.2	82.5	92.2	89.5	86.7

			70	70.2	67.5	64.8	74.4	71.7	69.0	78.7	76.0	73.2
Home	Champions	Champions	80	77.3	74.6	71.9	81.6	78.8	76.1	85.8	83.1	80.4
			90	84.4	81.7	79.0	88.7	86.0	83.3	92.9	90.2	87.5

**Equation 1 (example):** TotDisTea = 7.077 + 0.284 × 0 + (-0.079 × 0 + 0.486 × 0 – 0.633 × 1) + (0.153 × 1 + 0.004 × 0 + 0.751 × 0) + 0.424 × 65 – 0.271 × 41 + 0.712 × 55 = 62.2 km

Without the TotDisOpp predictor variable, the equation was the following, but this model had *R^2^_adj_* = .6429 and a prediction loss of 18.08%:

**Equation 2:** TotDisTea (km) = 24.82 + 0.273 × MatLoc1 + (0.072 × TeaLea2 + 1.000 × TeaLev3 – 0.227 × TeaLev4) + (0.186 × OppLev2 + 0.722 × OppLev3 + 0.637 × OppLev4) + 1.081 × EffPlaTim – 0.159 × BalPos

## DISCUSSION

The main aim of this study was to estimate two predictive linear regression models using the TotDisTea and TotDist21Tea by combining eight predictor variables. A total of 127 models were estimated from the all-possible regressions procedure for each outcome variable. The model with all predictor variables except MatOut was selected as the best model to predict the TotDisTea (*R^2^_adj_* = .82). The predictors TotDisOpp, EffPlaTim, and BalPos had a greater contribution to the TotDisTea outcome than the predictors OppLev, TeaLev, and MatLoc. The model to predict TotDis21Tea was not built because both the maximum model and the 126 derived sub-models had little predictive power (*R^2^_adj_* < .38). With the results, it could be concluded that theoretically, the interaction of teams in the football matches has also been verified in the conditional dimension, with a close relationship between the physical responses of both teams. From a practical point of view, practitioners could have the possibility to estimate physical performances of teams in matches when it has not been possible to obtain that outcome (e.g., TotDisOpp).

Football is a sport of interaction and, therefore, the performance of a team is dependent on the performance of the opponent [20]. Multidimensional proposals to characterize playing styles are increasing [21]. The unique way teams play means that the distribution of roles among players is specific, so individual dimensions (e.g., emotional, cognitive, affective, behavioural, social, and conditional) will be unequally demanded. For this reason, the same positions in different teams carry with them different conditional responses, that is, adjusted to the way the team competes [22], greater movement of the ball or models that enhance the exploration of a more direct game. A revealing aspect of this study was the effect of TotDisOpp on TotDisTea when the rest of the model’s predictors were held constant; the interpretation is as follows: for each km run by the opponent team, there is an expected increase in mean TotDisTea of 0.71 km, 95% CI [0.69, 0.74]. This would affect both positively (the need to run more) and negatively (forced to run less) whether the conditional profile of the opponent faced is higher or lower than the reference team. When the predictive model has been applied eliminating the variable TotDisOpp (equation 2) the model had *R^2^_adj_* = .64, and a level of prediction loss of 18.1% with respect to equation 1 (with TotDisOpp), which carries a great weight within the predictive model. For this reason, when one wants to assess the physical performance of a team, as can be deduced from this study, keeping in mind the physical performance of the opponent is essential.

The second predictive variable that most influenced the prediction of TotDisTea was EffPlaTim. More than a decade ago it was reported that in the effective playing time (EPT), the player accumulates the largest amount of physical demand, this percentage increasing as the running speed increases, and it can be close to 100% in the case of high-speed running [1]. On the other hand, nowadays, in professional football leagues, since the implementation of the Video Assistant Referee (VAR), there have been some changes, especially in total (TPT) and effective (EPT) playing time [23]. Although there was no significant effect in the technicaltactical dimension (e.g., passes, dribbles, crosses, shots, goals, corners, fouls, width, length, height, distance from the goalkeeper to their defence), physical performances in Spanish LaLiga teams had a slight decrease in the total distance covered (108.9 vs. 107.9 vs. 106.9 km) when VAR intervened (VAR0, VAR1 and VAR2, respectively). Probably, it is due to the decrease in EPT between VAR0 and VAR1 (52.5 vs. 51.5 min, respectively) and a slight increase in TPT in VAR2 compared to VAR1 and VAR0 (99.1 vs. 96.0 vs. 95.1 min, respectively).

Ball possession is one of the most studied variables in elite football, above all, in the attempt to associate it with success [5]. From the interpretation of the results, as shown in Table 4, the BalPos variable is the third of the variables in predictive importance of TotDisTea. For each minute of ball possession, a decrease in mean TotDisTea of -0.27 km, 95% CI [-0.29, -0.25] is expected. Usually, successful teams are those that have greater possession [5] and less accumulated distance [24, 25]; nevertheless, the methodology used in this type of research does not tell us whether the offensive phase is more or less physically demanding compared to the defensive phase. A recent study [8] tried to relate the effective time of the game (distinguishing the phases of possession and non-possession of the ball) with the locomotor response, from an intensity variable, meters per minute covered by the team. Two of the main conclusions of the study were that teams ran more per minute when players did not have the ball than when they did, and the distance accumulated per minute by the teams in ball possession does not correlate with the distance accumulated in the non-possession phase and vice versa [8]. Then, the physical response assessment needs information about the accumulated time of possession.

To assess the level of the teams, result indicators are usually used, such as the number of goals [5] or accumulated points [26], or the classification at the end of the championship [24], among others. In a previous study, carried out in the same Spanish league [5], some significant differences were observed (with a trivial effect size) in the physical performance of the teams in the upper half of the table compared to the last in the standings. Similarly, in the current study, the Europa League teams also had a greater physical response (increase of almost half a kilometre) with respect to the reference value taken from the relegation teams. By contrast, the groups of teams staying up and in the Champions League showed negative values with respect to the reference value; it can be interpreted that physical response was not a dimension that characterized them. It seems that each team tries to take advantage of its strengths (e.g., running or passing more than the opponent does) as their style of play. However, regarding the particularity of each match, the quality of the opponent shows a linear trend like that described in the literature [1, 10]; the greater the quality of the opponent (determined by their standing in the league) the greater is the locomotor activity demanded from the reference team. From the models proposed in the present study, unlike what was observed in a previous study [27] where the time of possession of the ball was not taken into account, it can be predicted that the teams having to face teams that are in the Champions League will increase the TotDisTea between 0.4 and 1.1 km. In any case, caution must be exercised when interpreting the results because the small differences in the final standings depend not so much on the team’s usual way of playing [21], but rather on the effectiveness of the team in shots on target [28]. Furthermore, exploring this study in other divisions (e.g., the Spanish second division of soccer) could be of interest [6].

The influence of match location on the physical response of players and teams is not new [1, 10]. The home teams usually covered a greater distance than away teams. A recent study found [8] that match location affected the distance covered at > 21 km/h but not the total distance accumulated per minute by the teams, running greater distance in matches played at home. In line with this, in the prediction model of our study, to play at home meant between 0.1 and 0.5 more km for teams. Several years ago, a review of the evidence for the hypothesized reasons for a home advantage was made [29]; crowd support, referee bias, psychological factors, travelling of away teams, familiarity with local conditions, territoriality and specific playing tactics were suggested as other possible influences. In any case, home teams seem to be forced to get a good result when they play at home.

MatOut was not included in the final predictive model. Nevertheless, it is known that match status has a big influence on the adopted strategy of the teams during a match, which accounts for the fact that players do not always use their maximal physical capacity for an entire match [2]. Changes in the match status create special needs within the team, being particularly affected the different positions in a playing system [30]. In line with this, Lago et al. observed that for every minute losing, players covered an extra metre of sprinting (> 19.1 km/h). However, while the team’s overall physical performance might not be greatly affected by changes in the match status, it could alter the distribution of locomotor demands among teammates. Losing status could increase the total distance and the distance covered at 14–21, 21–24 and > 24 km/h by defenders, while attacking players could increase the distances accumulated in these ranges of velocity during winning status [30]. The difference between the studies could be explained by two reasons: methodologically, the fact that the physical performance evaluated in the present study is limited exclusively to effective playing time; and conceptually, because although the outcome of the match has been classified as win, draw or loss, it is known that during matches there may have been changes in the match outcome, being able to have a different temporal distribution (% of time winning, losing and drawing).

One of the methodological limitations of the present study was to determine the minimum sample size required to conduct a multiple linear regression analysis; the general rule-of-thumb of *N* ≥ 50 + 8 × *p* was applied [31, 32], where *p* is the number of parameters of the maximum model. In our case, if the maximum model had 13 parameters, then this model had to be built with at least 50 + 8 × 13 = 154 observations. The reason for applying this rule was that no previous football studies were found that built a model to predict the total distance covered by teams. However, considering the results obtained in the present study (*R^2^_adj_* = .82; *M* and *SD* of TotDisTea = 78.3 and 6.3 km, respectively) and the four-step procedure proposed by Riley et al. [33, 34], future football studies will be able to more adequately calculate the minimum sample size required to build a linear regression model to predict the total distance covered by a team. A second limitation of the study concerns the levels set for some situational variables. Probably, distributing the match outcome according to minutes winning, losing or drawing could improve the degree of prediction of the model. In addition, the level of the team and its rivals, which has been established considering the ranking at the end of the season, does not take into account the variability of the position occupied by the teams on each match day of the championship. The third limitation is related to the idiosyncrasy of the players and teams in the way of task solving, that is, playing a football game. It would be interesting to know the weight carried by physical demand in each team performance, then apply this type of predictive model to each team in *LaLiga*.

## CONCLUSIONS

Our results highlight a number of variables that could explain physical workload in football players, and combinations of these variables could be used to develop a model for predicting (from a probabilistic viewpoint) the physical activity profile in competition. The main conclusions of the present study focus on the importance of the opponents in the physical performance when a team tries to resolve the task of playing a match. Apart from the opponent, effective playing and possession times are placed in a secondary position. Finally, with less importance, match location and match-up quality must be considered when planning to predict the total distance that will be covered by the team. The findings of this study suggest again [8] that an effective assessment of football performance at a behavioural level needs to incorporate both the different contextual variables (and their interactions) where the match has developed, and the particular strategic variables that the teams have proposed in the match according to the needs at each moment of the match.

## Data Availability

https://rhileno.shinyapps.io/TotDisTea_prediction/
